# Direct Electrical Current Reduces Bacterial and Yeast Biofilm Formation

**DOI:** 10.1155/2016/9727810

**Published:** 2016-03-17

**Authors:** Maria Ruiz-Ruigomez, Jon Badiola, Suzannah M. Schmidt-Malan, Kerryl Greenwood-Quaintance, Melissa J. Karau, Cassandra L. Brinkman, Jayawant N. Mandrekar, Robin Patel

**Affiliations:** ^1^Division of Clinical Microbiology, Department of Laboratory Medicine and Pathology, Mayo Clinic, Rochester, MN 55905, USA; ^2^Division of Biomedical Statistics and Informatics, Department of Health Sciences Research, Mayo Clinic, Rochester, MN 55905, USA; ^3^Division of Infectious Diseases, Department of Medicine, Mayo Clinic, Rochester, MN 55905, USA

## Abstract

New strategies are needed for prevention of biofilm formation. We have previously shown that 24 hr of 2,000 *µ*A of direct current (DC) reduces* Staphylococcus epidermidis* biofilm formation* in vitro*. Herein, we examined the effect of a lower amount of DC exposure on* S. epidermidis*,* Staphylococcus aureus*,* Escherichia coli*,* Pseudomonas aeruginosa*,* Propionibacterium acnes*, and* Candida albicans* biofilm formation. 12 hr of 500 *µ*A DC decreased* S. epidermidis*,* S. aureus*,* E. coli*, and* P. aeruginosa* biofilm formation on Teflon discs by 2, 1, 1, and 2 log_10_ cfu/cm^2^, respectively (*p* < 0.05). Reductions in* S. epidermidis*,* S. aureus*, and* E. coli* biofilm formation were observed with as few as 12 hr of 200 *µ*A DC (2, 2 and 0.4 log_10_ cfu/cm^2^, resp.); a 1 log_10_ cfu/cm^2^ reduction in* P. aeruginosa* biofilm formation was observed at 36 hr. 24 hr of 500 *µ*A DC decreased* C. albicans* biofilm formation on Teflon discs by 2 log_10_ cfu/cm^2^. No reduction in* P. acnes* biofilm formation was observed. 1 and 2 log_10_ cfu/cm^2^ reductions in* E. coli* and* S. epidermidis* biofilm formation on titanium discs, respectively, were observed with 12 hr of exposure to 500 *µ*A. Electrical current is a potential strategy to reduce biofilm formation on medical biomaterials.

## 1. Introduction

Biofilms are associated with a variety of persistent infections as a result of their propensity to form and grow on foreign bodies. Compared with planktonic forms, organisms in biofilms exhibit increased resistance to the host immune system and antimicrobial therapy [1]; for this reason, the management of biofilm-associated infections is challenging. Today, many of these infections are definitively managed using medical device removal, an intervention that is both costly and inconvenient [2].

Given that biofilm-associated infections are difficult to manage, prevention strategies are ideal [3]. Most preventive approaches utilize antimicrobials or antiseptics [4–8]; however, considering that biofilms can survive in the presence of high concentrations of antimicrobial agents, new prophylactic strategies are needed. Chemical and mechanical strategies such as silver or gallium ions, cationic molecules, and other disinfectants have been studied as coatings of indwelling devices [9–12]. Substances with antibiofilm activity, such as lactoferrin or synthesized chalcones [13–15], as well as low acoustic energy [16, 17] have shown some ability to prevent biofilm formation. None of these strategies has, however, solved the clinical challenge of biofilm-associated infections.

The initial step of biofilm formation on medical devices involves adhesion of organisms to medical implant surfaces by electrostatic forces which are largely repulsive, as both are negatively charged [18]. Direct current (DC) may augment repulsive electrostatic forces between organisms and medical implants [19–22]. In addition, DC may impact biofilm formation by changing physical conditions (e.g., temperature, pH) at the implant surface and through the accumulation of products of oxidative stress [20, 23–27].

Previous studies have demonstrated that DC exhibits bactericidal activity against established biofilms [20, 22, 23, 25, 28]. The bactericidal effect of DC against sessile cells suggests that this strategy may be useful to reduce biofilm formation [19]. In a previous study, we showed that 24 hours of 2,000 *µ*A DC reduced* S. epidermidis* biofilm formation [29]. Whether lower amperage of DC would also reduce biofilm formation and whether our findings with* S. epidermidis* generalize to other microorganisms are unknown.

The use of DC to reduce biofilm formation may provide a new strategy to prevent biofilm formation in clinical practice. It has the potential benefit of eliminating the use of traditional antimicrobials and therefore decreasing the risk of selecting resistance to these agents. Herein, we examined the effect of different amperages and delivery durations of DC in reducing formation of biofilms of five bacterial and one fungal species.

## 2. Materials and Methods

### 2.1. Microorganisms

*S. epidermidis* Xen 43 [30],* Staphylococcus aureus* Xen 30 [31],* Escherichia coli* (IDRL-7029, prosthetic hip infection clinical isolate),* Pseudomonas aeruginosa* Xen 5 [32],* Candida albicans* (GDH2346, mouth infection clinical isolate), and* Propionibacterium acnes* (IDRL-7676, prosthetic shoulder infection clinical isolate) were studied. The Xen strains were generous gifts of PerkinElmer Caliper Life Sciences (formerly Xenogen Corp., Waltham, MA); GDH2346 was from Drs. Jyotsna Chandra and Mahmoud Ghannoum (University Hospitals of Cleveland and Case Western Reserve University, Cleveland, OH).

### 2.2. Treatment Device

**Figure 1:**
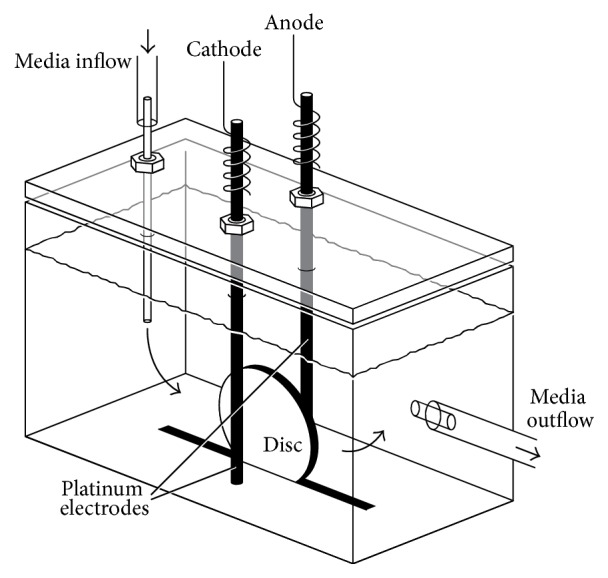
Setup of the treatment device. Electrodes are 3 mm from the disc.

Experiments were performed using polycarbonate channeled chambers designed and fabricated by the Mayo Division of Engineering (Figure 1). Each chamber contained a groove into which a 12.5 × 1 mm Teflon or titanium disc was inserted, positioned vertically. Cylindrical platinum electrodes, 1.5 × 55 mm, were placed in each chamber, 3 mm from the disc, with 1 cm of electrode extended above the chamber for the purpose of connecting the electrode to a current generator.

### 2.3. Electricity Generator

A power source (Keithley 2400 SourceMeter) or an 8-channel computer controlled current generator (designed by Mayo Division of Engineering) was used to deliver direct current (200 or 500 *µ*A).

### 2.4. *S. epidermidis*,* S. aureus*,* P. aeruginosa*, and* E. coli* Studies

Microorganisms were subcultured from frozen aliquots onto BBL*™* Trypticase*™* Soy Agar with 5% sheep blood plates (TSA II, Becton Dickinson Franklin Lakes, NJ) and incubated overnight at 37°C in 5% CO_2_. One colony was added to 3 mL of Trypticase soy broth (TSB) and grown for 1-2 hours at 37°C on an orbital shaker. The broth was adjusted to a 0.5 McFarland standard and added to a previously described semisynthetic medium [25] supplemented with 64 mL of 1% glucose and TSB (10%) to a final bacterial concentration of 10^3^ colony forming units (cfu)/mL.

A continuous flow (3 mL/hour) of the semisynthetic medium containing 10^3^ cfu/mL test organism was delivered to the polycarbonate treatment chambers containing Teflon or titanium discs. After 2 hours (for Gram-negative bacilli) or 4 hours (for Gram-positive cocci), the semisynthetic medium containing the test organism was changed to a phosphate buffer (12.78 mg Na_2_HPO_4_, 6.15 mg KH_2_PO_4_, and 19.2 mg glucose in 1000 mL sterile water) without bacteria, also flowing at 3 mL/hour.

DC (0, 200, or 500 *µ*A) was delivered (starting at the same time that semisynthetic medium with bacteria flow started) for either 4, 8, 12, 16, 20, or 24 hours of a 24-hour period when testing 500 *µ*A DC, or 12, 24, 36, or 48 hours of a 48-hour period when testing 200 *µ*A DC, with 0 *µ*A controls tested at each time point. Testing was performed at 37°C for Gram-positive cocci and at room temperature for Gram-negative bacilli.

After 24 hours when using 500 *µ*A or 48 hours when using 200 *µ*A, discs were aseptically removed from the test chambers, planktonic organisms rinsed off by gently dipping the discs into sterile saline, and the discs placed into sterile tubes containing 1 mL of sterile saline. Biofilm organisms were removed by vortexing and sonication in an ultrasound bath (40 KHz, 320 mW/cm^2^) for 5 minutes [33]. Suspensions of disaggregated biofilms were quantitatively cultured. The medium that remained in the chamber (planktonic organisms) after 24 or 48 hours was also quantitatively cultured. Each test was done in triplicate for each microorganism. Biofilm results were expressed as log_10_ cfu/cm^2^; planktonic results were expressed as log_10_ cfu/mL.

### 2.5. *C. albicans* Studies

The method described above was performed with the following modifications.* C. albicans* was subcultured from frozen aliquots and incubated for 48 hours at 30°C in room air, only 0 and 500 *µ*A DC for 24 and 48 hours were tested, semisynthetic medium was changed to phosphate buffer after 4 hours, and experiments were conducted at room temperature.

### 2.6. Anaerobic Studies

For* P. acnes*, experiments were performed as stated above with the following modifications. The organism was subcultured from frozen aliquots and incubated for 72 hours at 37°C under anaerobic conditions, only 0 and 500 *µ*A DC for 24 and 48 hours were tested, semisynthetic medium was changed to phosphate buffer after 4 hours, and experiments were conducted at 37°C in an anaerobic chamber (Coy Laboratory Products, Grass Lake, MI). In addition to performing experiments under aerobic conditions,* S. epidermidis* experiments were run under anaerobic conditions using 0 and 500 *µ*A DC for 24 hours.

### 2.7. Titanium Disc Studies

We compared the difference between* S. epidermidis* and* E. coli* biofilm formation on titanium discs using 0, 200, and 500 *µ*A of DC for 12 and 24 hours and the treatment device and methods described above.

### 2.8. Statistical Methods

Reductions in biofilm or planktonic cells were calculated comparing quantitative cultures of discs or surrounding fluid in chambers exposed and not exposed to electrical current. Statistical analyses were performed using SAS software (SAS Institute, Inc., Cary, NC). A one-way analysis of variance was performed with each current delivery strategy and no current delivery using the Wilcoxon rank sum test to determine if electricity reduced biofilm formation. All tests were two-sided; *p* values < 0.05 were considered statistically significant.

## 3. Results

### 3.1. *S. epidermidis*,* S. aureus*,* P. aeruginosa*, and* E. coli* Studies

**Figure 2:**
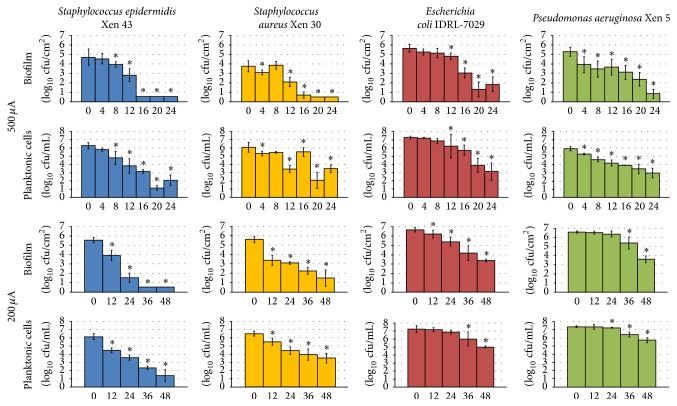
Results of quantitative cultures of* Staphylococcus epidermidis*,* Staphylococcus aureus*,* Escherichia coli*, and* Pseudomonas aeruginosa* biofilms on Teflon discs and associated planktonic cells with 200 and 500 *µ*A DC started at the time of bacterial seeding of the discs. The *x*-axis shows hours of DC exposure. The *y*-axis shows results of quantitative cultures in log_10_/cm^2^ for biofilm and log_10_/mL for planktonic cultures. The 0 *µ*A controls that were tested at each time point were combined for graphical purposes. ^*∗*^Statistical significance compared to exposure to no current (*p* < 0.05).

Time- and dose-dependent reductions in biofilm formation on Teflon discs were observed for* S. epidermidis*,* S. aureus*,* E. coli*, and* P. aeruginosa*, using 500 and 200 *µ*A (Figure 2). For* S. epidermidis*, a 1 log_10_ cfu/cm^2^ reduction in biofilm formation was observed starting at 8 hours of exposure to 500 *µ*A, with a 4 log_10_ cfu/cm^2^ reduction observed after 16 hours of exposure to 500 *µ*A or 24 hours of exposure to 200 *µ*A. For* S. aureus*, there were 2 log_10_ cfu/cm^2^ reductions in biofilm formation with 12 or more hours of exposure to 200 and 500 *µ*A. For* E. coli*, there were 1 and 4 log_10_ cfu/cm^2^ reductions in biofilm formation with 12 and 24 hours of exposure to 500 *µ*A, respectively; a similar but smaller effect was observed with 200 *µ*A, with a 4 log_10_ cfu/cm^2^ reduction observed with 48 hours of exposure. For* P. aeruginosa*, a 1 log_10_ cfu/cm^2^ reduction in biofilm formation was observed with 4 hours of exposure to 500 *µ*A or 36 hours of exposure to 200 *µ*A, with a 4 log_10_ cfu/cm^2^ reduction being observed after 24 hours of exposure to 500 *µ*A. Overall, significant reductions in biofilm formation were observed using 500 *µ*A for at least 12 hours (*p* = 0.0495) and 200 *µ*A for at least 36 hours (*p* < 0.05) for all four bacteria studied. Significant differences in amounts of planktonic cells were observed using 500 *µ*A for at least 12 hours (*p* = 0.0495) and 200 *µ*A for at least 36 hours (*p* = 0.0495) for all four bacteria studied.

Since DC reduced* S. epidermidis*,* S. aureus*,* E. coli*, and* P. aeruginosa* biofilm formation on Teflon discs, we next tested whether this effect would be observed with yeast and an anaerobic bacterium on Teflon discs, as well as with* S. epidermidis* and* E. coli* on titanium discs.

### 3.2. *C. albicans* Studies

**Figure 3:**
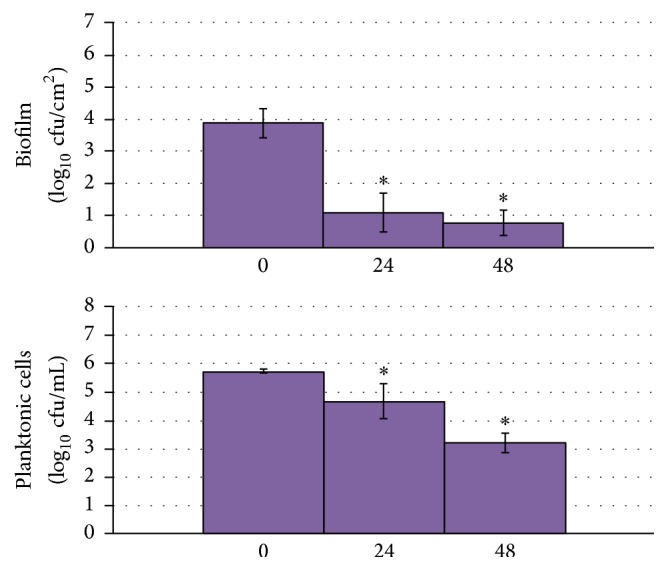
Results of quantitative cultures of* Candida albicans* biofilms on Teflon discs and associated planktonic cells with 500 *µ*A DC exposure started at the time of candidal seeding of the discs. The *x*-axis shows hours of DC exposure. The *y*-axis shows results of quantitative cultures in log_10_/cm^2^ for biofilm and log_10_/mL for planktonic cultures. The 0 *µ*A controls that were tested at each time point were combined for graphical purposes. ^*∗*^Statistical significance compared to exposure to no current (*p* < 0.05).

A 3 log_10_ cfu/cm^2^ reduction in* C. albicans* biofilm formation on Teflon discs was detected after 24 hours of exposure to 500 *µ*A DC (Figure 3).

### 3.3. Anaerobic Studies

**Figure 4:**
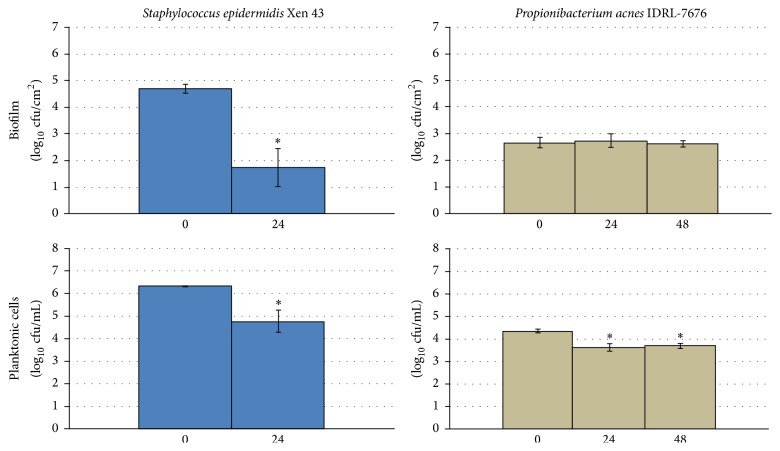
Results of quantitative cultures of* Staphylococcus epidermidis* and* Propionibacterium acnes* biofilms on Teflon discs and associated planktonic cells with 500 *µ*A DC exposure started at the time of bacterial seeding of the discs for experiments performed under anaerobic conditions. The *x*-axis shows hours of DC exposure. The *y*-axis shows results of quantitative cultures in log_10_/cm^2^ for biofilm and log_10_/mL for planktonic cultures. For* P. acnes*, the 0 *µ*A controls that were tested at each time point were combined for graphical purposes. ^*∗*^Statistical significance compared to exposure to no current (*p* < 0.05).

There was no reduction in* P. acnes* biofilm formation with 48 hours of exposure to 500 *µ*A DC (Figure 4), although there was a 1 log_10_ cfu/mL reduction in planktonic* P. acnes* with exposure to 500 *µ*A DC for 24 hours. A 3 log_10_ cfu/cm^2^ reduction in* S. epidermidis* biofilm was observed with 24 hours of exposure to 500 *µ*A (Figure 4).

### 3.4. Titanium Disc Studies

1 and 2 log_10_ cfu/cm^2^ reductions in* E. coli* and* S. epidermidis* biofilm formation on titanium discs, respectively, were observed with 12 hours of exposure to 500 *µ*A (*p* = 0.0495). A 1 log_10_ cfu/cm^2^ reduction in biofilm formation was observed for both* E. coli* and* S. epidermidis* on titanium discs with 24 hours of exposure to 200 *µ*A (*p* = 0.0495). The overall magnitude of the effect observed with titanium and Teflon discs was similar for both bacteria, although* E. coli* (means of 5 versus 6 log_10_ cfu/cm^2^, *p* = 0.0009) but not* S. epidermidis* (*p* = 0.0765) formed slightly less biofilm on untreated titanium than Teflon discs.

## 4. Discussion

Results of these studies demonstrate that DC reduces* Staphylococcus* species,* E. coli*,* P. aeruginosa*, and* C. albicans* biofilm formation. Since these microorganisms are frequently involved in biofilm-associated infections, these findings are of potential clinical interest.

Although our results are consistent with previous data showing a bactericidal effect of DC against sessile and planktonic cells [22, 25, 26, 34], previous studies have focused on treatment of established biofilms. Our results provide evidence that DC can reduce biofilm formation by staphylococci, Gram-negative bacilli, and* Candida* species. We observed both dose- and time-dependent responses using the strategy studied. Overall, a reduction in biofilm formation was measureable within 12 hours of application of 500 *µ*A DC; when applying 200 *µ*A of DC, an effect was observed after 36 hours of current application. The same effect was observed for planktonic bacteria and yeast.

DC may reduce the formation of biofilms by preventing adherence of bacterial cells to surfaces [20], through augmentation of the noncovalent forces between organisms, and in our study Teflon and titanium discs. However, the decrease in the observed planktonic cell population suggests that there may be additional active mechanisms. Direct damage from DC to bacteria or yeast by electroporation and/or production of reactive oxygen species, as well as generation of other toxic substances, has been proposed. Chlorine has been identified as a toxic substance that plays a role in the bactericidal effect of electrical current against established biofilms [27]. The absence of an effect against* P. acnes* biofilm formation may be explained by the involvement of reactive oxygen species in the mechanism underlying the antibiofilm activity of electrical current. The contribution of reactive oxygen species to this process is also supported by the decreased effect observed under anaerobic conditions with* S. epidermidis*.

Electrode composition may impact the activity observed. We used platinum electrodes to avoid corrosion associated with stainless steel electrodes [28]. Differences in bactericidal effect have been described when using different electrode materials; we observed less antibiofilm effect when using stainless steel compared with platinum electrodes (data not shown). It is possible that platinum complexes contributed to the effect observed [35].

Although most of our experiments were performed using Teflon discs, we demonstrated a similar effect using titanium discs, which is of clinical relevance since titanium is used in the construction of orthopedic implants.

Further investigation is needed to determine the appropriate dose and time of administration of DC for reduction of biofilm formation. Future work could explore the capacity of cells to adhere to a surface that has been previously exposed to electrical current and intermittent DC administration. Ultimately,* in vivo* studies will be required to address efficacy and safety.

Overall, our results demonstrate that biofilm formation can be reduced using low dose DC. Potentially, this strategy could be used during surgery to prevent early infection and contamination of newly implanted foreign bodies.
